# RF energy harvesters for wireless sensors, state of the art, future prospects and challenges: a review

**DOI:** 10.1007/s13246-024-01382-4

**Published:** 2024-01-17

**Authors:** Nasir Ullah Khan, Farid Ullah Khan, Marco Farina, Arcangelo Merla

**Affiliations:** 1 Department of Engineering and Geology, Università degli Studi “G. d’Annunzio” Chieti – Pescara, Pescara, 65127 Italy; 2 Department of Mechatronics Engineering, University of Engineering and Technology, Peshawar, Peshawar, 25000 Pakistan; 3 Department of Information Engineering, Università Politecnica delle Marche, Ancona, 60131 Italy

**Keywords:** RF energy harvesting, Biocompatible RF antennas, RF-powered medical sensors, Rectifier circuit, Wireless sensors

## Abstract

The power consumption of portable gadgets, implantable medical devices (IMDs) and wireless sensor nodes (WSNs) has reduced significantly with the ongoing progression in low-power electronics and the swift advancement in nano and microfabrication. Energy harvesting techniques that extract and convert ambient energy into electrical power have been favored to operate such low-power devices as an alternative to batteries. Due to the expanded availability of radio frequency (RF) energy residue in the surroundings, radio frequency energy harvesters (RFEHs) for low-power devices have garnered notable attention in recent times. This work establishes a review study of RFEHs developed for the utilization of low-power devices. From the modest single band to the complex multiband circuitry, the work reviews state of the art of required circuitry for RFEH that contains a receiving antenna, impedance matching circuit, and an AC-DC rectifier. Furthermore, the advantages and disadvantages associated with various circuit architectures are comprehensively discussed. Moreover, the reported receiving antenna, impedance matching circuit, and an AC-DC rectifier are also compared to draw conclusions towards their implementations in RFEHs for sensors and biomedical devices applications.

## Introduction

Taking into account the advancements in micro-electromechanical systems (MEMS) and nanotechnology, the applications of low-power sensors and biomedical devices are extended to areas, such as communication, automation, manufacturing, transportation, aviation, defense, and health. Due to their significant characteristics, for example, low power consumption, light weight, small size, fast response and high precision, such devices are being favored [1]. The global market value of automotive MEMS sensors is reported approximately US $27.23 billion in 2023 and is assumed to extend up to US $36.53 billion till 2028 to increase with the compound annual growth rate (CAGR) measuring 6.05% during the years 2023 to 2028 [2]. Similarly, the global market size of digital health which mainly uses biomedical devices is reported US $245.3 billion in 2023 and is presumed to increase with a CAGR of 18.6% during the period of 2024 to 2030 [3]. However, the major concern associated with such nano and micro-scale devices is their dependency entirely on battery for their operation [4]. In the past decade, researchers have proposed and developed several energy harvesting techniques which are capable of operating MEMS-based wireless sensor nodes (WSNs) and low-power IMDs. Different forms of ambient energies are present in the environment, such as vibration [5], acoustic [6], thermal [7], wind [8], and solar [9] which can be efficiently harvested using corresponding harvesters. The power consumption of sensors, wearable gadgets and biomedical devices is listed in Table 1.

**Table 1:** Power consumption specifications of wireless sensors and implantable devices

Low power sensors & IMDs	Cardiac pacemaker	Cochlear implant	Electronic watch	Smoke alarm	Glucose sensor	Temperature sensor
References	[9–11]	[12–14]	[15]	[15]	[16]	[17]
Voltage required (V)	2–5	1.5–5	1.5–3	3–12	3–5	2.1–3
Power required (µW)	< 100	600–40,000	1	1000	3	400
Power level	Ultra-low	Low	Ultra-low	Low	Ultra-low	Low

## Ambient RF energy

The pervasive use of wireless communication has led to the abundant presence of RF energy residue in the environment. In wireless communication, electromagnetic signals with a certain frequency are propagated by a transmitter into the free space while the receiver collects these electromagnetic signals. The distance (R) from the transmitter to the point of receiving the transmitted signal can be classified into three fields. Reactive near field, radiating near field and far field. The reactive near field and radiating near field are in close vicinity of the transmitting antenna, and the electric (E) and magnetic (B) fields are not in phase exactly, also field distribution can`t be approximated as it is highly dependent on distance and direction of the transmitter. The far-field propagates till infinity, the E and B fields are in phase and the field distribution can be approximated. Wireless power transfer (WPT), particularly in the context of medical implants, concentrates on the reactive and radiating near field [18], where the proximity between transmitter and receiver is kept minimal to mitigate misalignment issues and adhere to specific absorption rate (SAR) restrictions. Similarly harvesting RF energy from the surroundings, the signals are typically acquired in the far field of the transmitter. The three regions of a transmitting antenna can be approximated by the following models.

For the reactive near field, the distance:

1 $$R<0.62 \sqrt{\frac{{D}^{3}}{\lambda }}$$

However, for radiating near field it is

2 $$0.62 \sqrt{\frac{{D}^{3}}{\lambda }}<R<\frac{{2D}^{2}}{\lambda }$$

And, for far field

3 $$R > \frac{{2D}^{2}}{\lambda }$$

Where D is the maximum linear dimension of antenna device while λ represents wave-length of the electromagnetic waves.

The RF spectrum is the part of the electromagnetic spectrum that ranges from very high frequencies (VHF) to extremely high frequencies (EHF) i.e. 3 kHz to 300 GHz. All the familiar transmission systems utilize some part of the RF spectrum to transmit the signals to the receiver end. Prominent transmission systems including FM radio (87.5 MHz–108 MHz), VHF and UHF DTV, GSM-900, GSM-1800, UMTS-3G, LTE-4G, WIFI, Bluetooth, ISM band (2.4 – 2.5 GHz) and 5G extensively employ the RF spectrum for broadcasting and communication purposes [19]. The quantity and availability of RF energy residue in the environment depends on the consumption of a specific band for wireless communication.

**Fig. 1:**
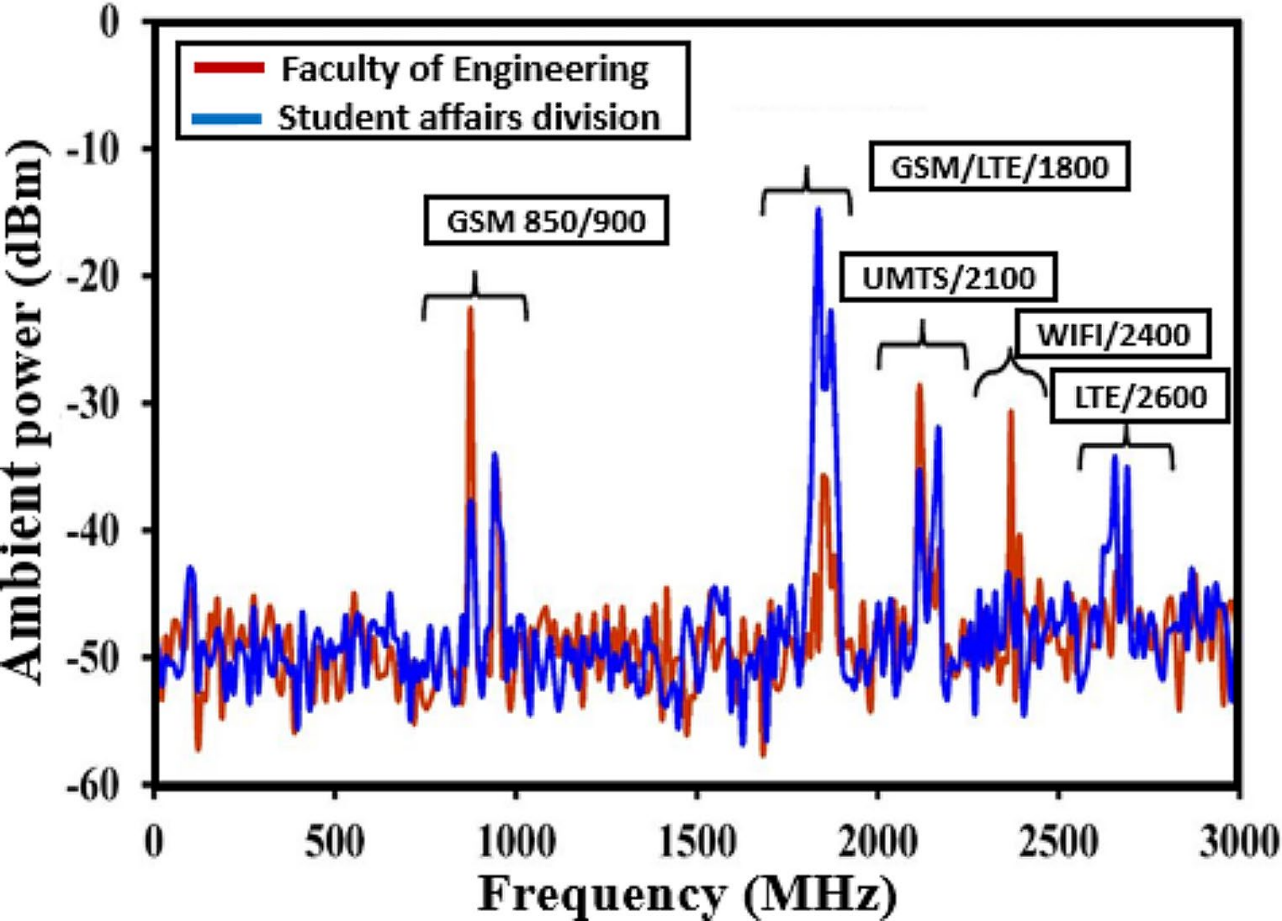
Input RF power densities estimated at MMU Cyberjaya, Malaysia, reproduced from Muhammad et al. [20], licensed under creative commons

Figure 1 illustrates a survey data, including received RF power from various transmitters, collected and analyzed using a 6 GHz spectrum analyzer at the Faculty of Engineering, MMU Cyberjaya, Malaysia [20]. The findings indicate that GSM-900 and GSM-1800 have reported maximum received power levels of -21.2 dBm and − 15.3 dBm, respectively. Similarly, Table 2 presents the RF power densities originating from various RF ambient sources in a survey conducted in London [21], which also revealed GSM-900 and GSM-1800 boast the highest power densities among these ambient RF sources. This suggests that GSM-900, GSM-1800, and UMTS-3G stand out as the primary contenders for RFEH, as they exhibit notably the highest RF power densities.

**Table 2:** RF power densities of various ambient sources near station, London [21]

RF source	Transmitting Frequency (MHz)	Power density (nW/cm^2^)
DTV	470–610	0.89
GSM900 (MT)^1^	880–915	0.45
GSM900 (BT)^2^	920–960	36
GSM1800 (MT)	1710–1785	0.5
GSM1800 (BT)	1805–1880	84
3G (MT)	1710–1785	0.46
3G (BT)	2110–2170	12
Wi-Fi	2400–2500	0.18

^1^MT Mobile transmitter, ^2^BT Base transmitter

**Fig. 2:**
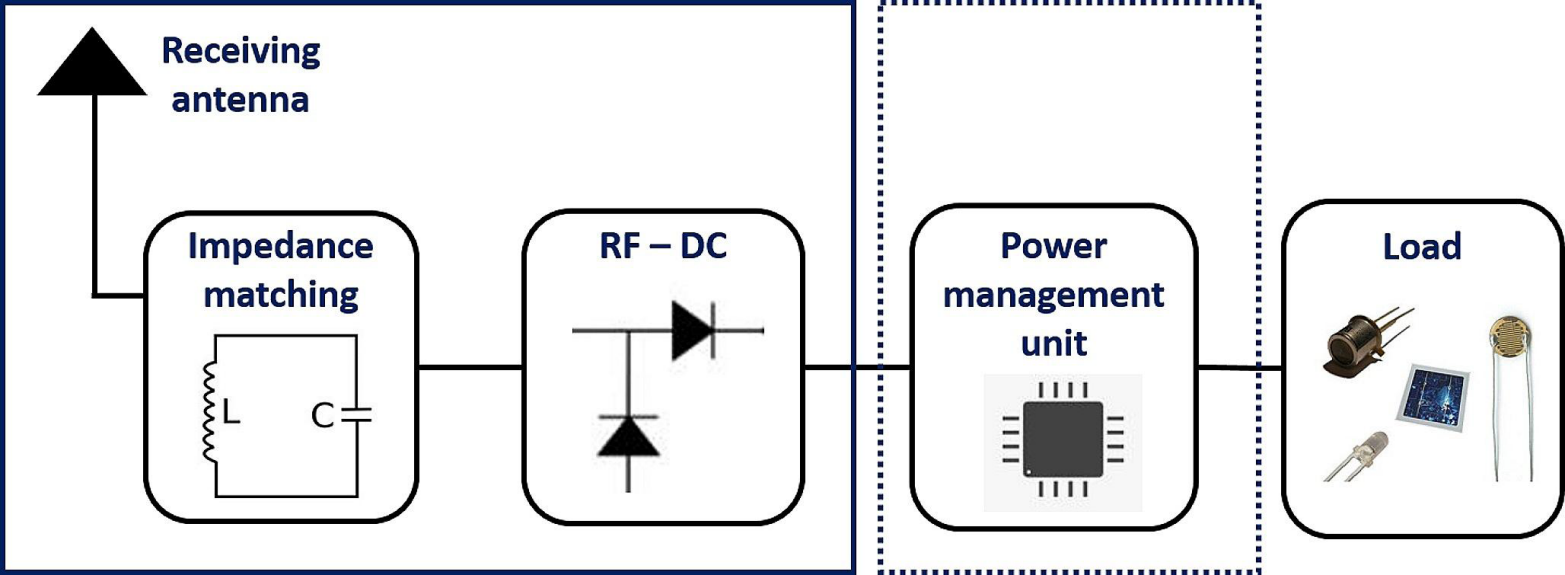
Schematic of a general RFEH system

With the enormous use of RF signals for wireless communication and technology, RFEH has gained extensive interest due to ambient RF energy residue present in the surrounding. RFEH is an advent, to convert ambient RF signals into useful electrical energy [22]. Major RF energy sources are mobile phone base stations, television/radio broadcasters, wireless fidelity (Wi-Fi) signal transmitters, mobile phones, Bluetooth and any source that transmits RF signals of certain frequency present in the surrounding [23]. Figure 2 represents schematic of general RFEH system. The key elements of an RFEH include the following components: a receiving antenna, responsible for capturing RF signals emitted by an RF source; an impedance matching circuit, designed to optimize power harvesting by aligning the impedance of the receiving antenna with the rectifier; and the AC-DC rectifier, which converts the collected signals into usable electrical power.

Depending on its applications, an RFEH may consist of a voltage booster or regulator at the output end to boost up the output voltage level for specific applications at the cost of size and efficiency of the harvester. Some RFEHs are developed with a power management unit (PMU) with the intent to provide continuous and stable power delivery to the load but in this case, the PMU may also require an alternative power source to RFEH. However, the addition of complex circuitry of PMU enlarges the size, increases power losses, and reduces the overall efficiency of the device. Table 3 offers an insight into prior research conducted in the realm of RFEHs. The reported review articles shed light on the prevailing patterns, approaches to implementation, and architectural designs employed within the RFEH domain. In contrast to earlier review articles, this study places its emphasis on strategies for miniaturized antennas, current trends, forthcoming opportunities, and the challenges that lie ahead within the field of RFEHs, particularly in the context of their application in low-power sensors and implantable devices.

**Table 3:** List and comparison of recent reviews on the state of the art of RFEH system

Ref.	Year	Review focused on	Remarks/summary
[24]	2013	Compact antennas design for RFEH	A detailed overview of various antenna designs has been discussed with the objective of harmonic rejection, polarization, re-configurability, and size miniaturization. Moreover, a comparison of adopted techniques for selecting compact antennas has also been presented.
[25]	2014	Architecture of wireless networks with RFEH capability	Circuit design and implementation of wireless networks based on RFEHs have been presented. Issues related to circuit design and development of such networks have been explained.
[26]	2015	Rectifiers circuitry for RFEH	Various CMOS on-chip rectifiers for the RFEHs have been discussed. Pros and cons of each topology have been studied comprehensively for different applications.
[27]	2017	Multiband antennas and implementation for RFEH	Existing multiband and broadband antennas for wireless EHs have been discussed. Comparison of technologies and novel development of broadband antennas have been proposed for dynamic input power level
[28]	2019	Wireless EH for wearable devices	Classification of various wireless energy techniques have been explained for wearable devices. Key challenges in the development of wearable devices have been highlighted for telemedicine’s application
[29]	2019	RFEH in 5G context	5G landscape and its associated environment is reviewed, also delved into the research trends and limitations concerning RFEHs within the realm of 5G technologies.
[30]	2020	Overall system efficiency in RFEH	Broad overview of various parameters involved in main blocks of RFEHs has reviewed. In order to enhance the harvester’s efficiency and minimize power consumption, a thorough analysis and explanation of these components are provided.
[31]	2022	Design methodology of RFEH for ultra-low power application	Qualitative and quantitative analysis of RFEH circuit architecture has been performed. Design challenges and considerations of circuitry have been critically discussed.
[32]	2022	Antenna design and fabrication challenges for RFEH	Existing compact antennas with features, such as, low profile, multiband, and circular polarization have been overviewed. Design and fabrication challenges of current antennas with future improvements have been presented.
[23]	2023	Power enhancement of WSNs by RFEH	Presented the challenges related to power conversion efficiency at low power and potential future development. The performance of various rectifier circuits and impedance-matching circuit for different loads has been studied.
This work	2023	Recent trends and advancement in design of RFEH	Overview of recent developments in the design of miniaturized receiving antennas, matching circuits, and recent trends in rectifier design. Focuses on the challenges and provide a guideline for the architecture and performance optimization of RFEHs for low power sensors and implantable are comprehensively discussed.

The effectiveness and optimization of a RFEH hinge on the efficiency and performance of individual modules, these modules can be integrated to collectively improve the overall efficiency. To achieve maximum overall efficiency in RFEH, it is imperative to set specific objectives for improving the performance of each module based on the application’s requirements. Several critical factors contribute significantly to the overall efficiency of RFEH. Firstly, the choice of a receiving antenna that aligns with the application’s dimensions and selects frequencies according to the available RF energy in the environment, considering its performance metrics like gain and efficiency, plays a pivotal role in achieving overall efficiency in RFEH. Similarly, the implicit implementation of an impedance matching circuit between the antenna and rectifier is instrumental in minimizing matching losses, further contributing to the overall effectiveness of RFEH. Lastly, the selection of an appropriate AC-DC rectifier constitutes a key module in realizing the overall goal of enhancing efficiency and effectiveness in RFEH. These aspects collectively determine the success and efficiency of the RFEH system.

## Receiving antenna of an RFEH

The receiving antenna (RA) plays an essential and pivotal role within RFEH, serving as the primary component for collecting residual RF energy from the surroundings. By the Friis transmission model [29], power concentrated at the node of RA can be represented as.

4 $${P}_{rx}={P}_{tx}{ G}_{tx} {G}_{rx}{\left(\frac{c}{4\pi f{D}_{r}}\right)}^{2}$$

The received power depends on transmitted power *Ptx*, gain of TA *Gtx*, gain of RA *Grx*, speed of light *c*, radio wave frequency *f*, and transmission distance *Dr* between TA and RA. It is fair to say that RA with the highest possible gain can be the desired choice for the RFEHs, however, the design of RA begins with the selection of an appropriate frequency band which may appraise key factors, such as availability of RF energy residue, region of installation, type of application and size of RFEH [33].

This review concentrates on the progress of RFEHs in the context of their utilization in low-power wireless sensors and implantable devices. Additionally, it delves into the recent advancements in the design of compact, low-profile antennas. The design of miniaturized antennas for RFEH has garnered significant attention in research circles over the past decade, primarily owing to its relevance in powering low-power sensors and implantable devices. In the literature, various antennas, monopoles [20, 34], dipoles [35–37], loops [38, 39], patches [18, 40–42], and planar inverted F [43–45] have been developed with single [18, 39, 40], dual [33, 34, 37], multi [45, 46], wideband [38, 48] and array [43, 47] topologies. Standard designing and simulation techniques have been developed and adopted over the past decade to improve the performance of RA. Further, validated approaches, such as reconfiguration, circular polarization, arrays, and meta-surfaces have the possibility to enhance the RA performance in terms of RFEH for low-power devices.

**Fig. 3:**
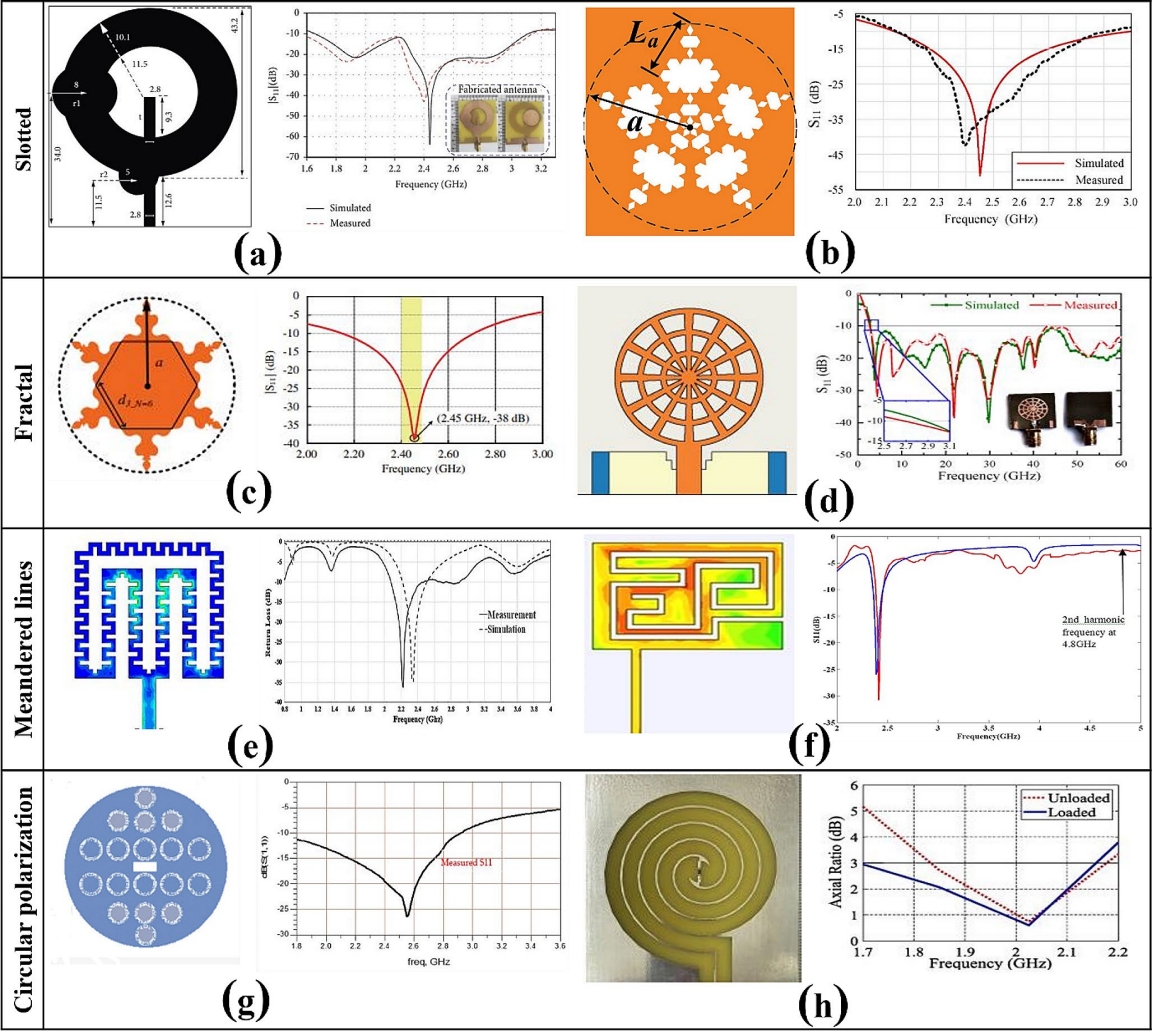
Miniaturized geometries and results of various RAs developed for RFEH: **a** and **b** slotted wideband antennas [50, 51], licensed under creative commons & Copyright (2018), with permission from Elsevier; **c** and **d** fractal wide and multiband antennas [52, 53], reproduced courtesy of The Electromagnetics Academy; **e** and **f** meandered lines wide and multiband antennas [54, 55], Copyright (2018 & 2016), with permission from Elsevier; **g** and **h** circular polarized ultra-wideband antennas [56, 57], licensed under creative commons & Copyright (2023), with permission from Elsevier

Arrawatia et al. [49] designed a single band GSM-900 square patch RA with a high gain of 9.1 dB and tested to harvest energy from a base station. The developed antenna was capable of harvesting 2.78 V DC with 5 dBm power at 10 m away from the base station. Planar Patch antennas which come with various geometries (rectangular, circular & elliptical, etc.) have remained a preferred choice for RFEHs over the years. This popularity is attributed to their advantages, including compact size, low profile, and easy design and manufacturing processes. However, it’s worth noting that they also exhibit limitations, including narrow bandwidth and low gain as highlighted by Patil and Gahankari [42]. To address these challenges and improve the capabilities of harvesting power on a broader scale, efforts have been directed towards the development of multi and wideband RAs and transforming the conventional into distinct geometrical configurations, such as, slotted [50, 51], fractal [52, 53], meandered lines [54, 55] and circularly polarized [56, 57] as shown in Fig. 3.

Muhammad et al. [50] developed circular patch RA, two circular and rectangular slots made the RA best choice for RFEH by achieving wideband and high gain of 2.7 dBi at 2.400 GHz with 2800 mm^2^ compact size. Similarly, rectangular patch RA is reported for RFEH [51], with a fractal-shaped slot in the patch enabling the RA of 32.5% reduction in overall size with a measured gain of 2.4 dBi at 2.45 GHz. Shi et al. [52] presented a miniaturized, compact, and unique fractal wideband antenna to harvest Wi-Fi signals. This unique geometry results in improving the gain (2.2 dBi), reflection coefficient (-38 dB) with miniaturized size of 1444 mm^2^. Likewise, super wideband fractal and miniaturized RA is reported [53], to target RF energy harvesting from 5G to 6G wireless communication. The developed RA exhibits a low profile of 400 mm^2^ with a high gain of 6 dBi for the desired frequency band. To attain compact RA dimensions, meandered lines techniques are commonly utilized in literature. For example, Celik and Kurt [54] presented a broadband E-shaped RA based on meandered line approach, with the applied approach the RA best suited for RFEH by achieving broadband, high efficiency (99.4%), high gain (3.78 dBi), and size reduction of approximately 23%. Also dual-band rectangular patch RA for RFEH is reported [55], by adopting meandered line techniques the RA achieved the desired frequency bands and miniaturized size (1750 mm^2^) with a characteristic gain of 2.8 dBi at 2.4 GHz. Since the exact location and direction of received signals are unpredictable, circular polarization (CP) for RA has been highly favored recently for RFEH applications. Sabban [56] proposed a novel wideband CP antenna for 5G technologies, effective size (990 mm^2^) and gain (8.3 dBi) were improved by using metamaterial with an antenna`s efficiency of 95%. Similarly, Jalali et al. [57] presented, CP multiband RA for RFEH in GSM-900, GSM-1800, and UMTS-3G frequency bands. The reported RA presents a better choice for RFEH by achieving 96% efficiency with a maximum gain of 5.93 dBi.

Table 4 provides a compilation of various antennas along with their specifications, focusing device geometry, dimensions, resonant frequencies, and gain characteristics. Reducing the physical dimensions of an antenna while simultaneously elevating its gain can prove to be a demanding endeavor, given that these aims frequently conflict due to the inherent physical characteristics of antennas. Nevertheless, several methods and design approaches exist to strike a harmonious balance between size reduction and gain enhancement. These include incorporating loading coils, slots, meandering structures, fractal geometries, utilizing high dielectric materials, and employing arrays of antenna elements.

**Table 4:** Specifications of reported miniaturized receiving antenna for RFEHs

Ref	Antenna geometry	Band type	Device dimension (mm^2^)	Resonating frequency (MHz)	Gain (dBi)
[39]	Rectangular loop	Single	1200	868	0.65
[41]	Microstrip patch	Single	447^1^	2450	7.69
[54]	Fractal patch	Single	1444	2450	2.2
[34]	Printed patch	Dual	9600	900, 1800	1.8, 2.06
[20]	Inverted F- monopole	Dual	2500	900, 1800	0.62, 2.36
[37]	Folded dipole	Dual	1200	915, 2450	1.87, 4.18
[38]	Four port loop	Dual	9273	915	1.3
[51]	Meandered lines rectangular patch	Dual	1750	1740, 2450	2.1, 2.6
[52]	Meandered lines E-shaped patch	Broad	3600	2200–2500	3.78
[42]	Microstrip patch	Wide	6400	1600–2450	6.28
[50]	Slotted circular patch	Wide	2800	1640–3150	2.7
[55]	Ring fractal patch	Wide	400^1^	3220–13,560	6.0
[58]	Dual-Dipole	Wide	10,000	1400–2950	9.9

^1^patch size

## Matching circuit of an RFEH

Achieving optimal RF power transmission necessitates the presence of an Impedance Matching Circuit (IMC) positioned between the RA and the rectifier. An IMC is a circuit or network designed to facilitate maximum power transfer from the source while minimizing signal reflection from the load. Typically, the impedance of receiving antennas remains fixed, often at 50 Ω, while the impedance of rectifier circuits varies based on the frequency and characteristics of the components, such as diodes and transistors, utilized within the rectifier circuit. The IMC serves the crucial purpose of adjusting the impedance of the rectifier circuit through optimization to align it with the impedance of the antenna.

5 $$ \Gamma ={S}_{11}= \frac{{Z}_{REC- }{Z}_{ANT}}{{Z}_{REC+ }{Z}_{ANT}}$$

**Fig. 4:**
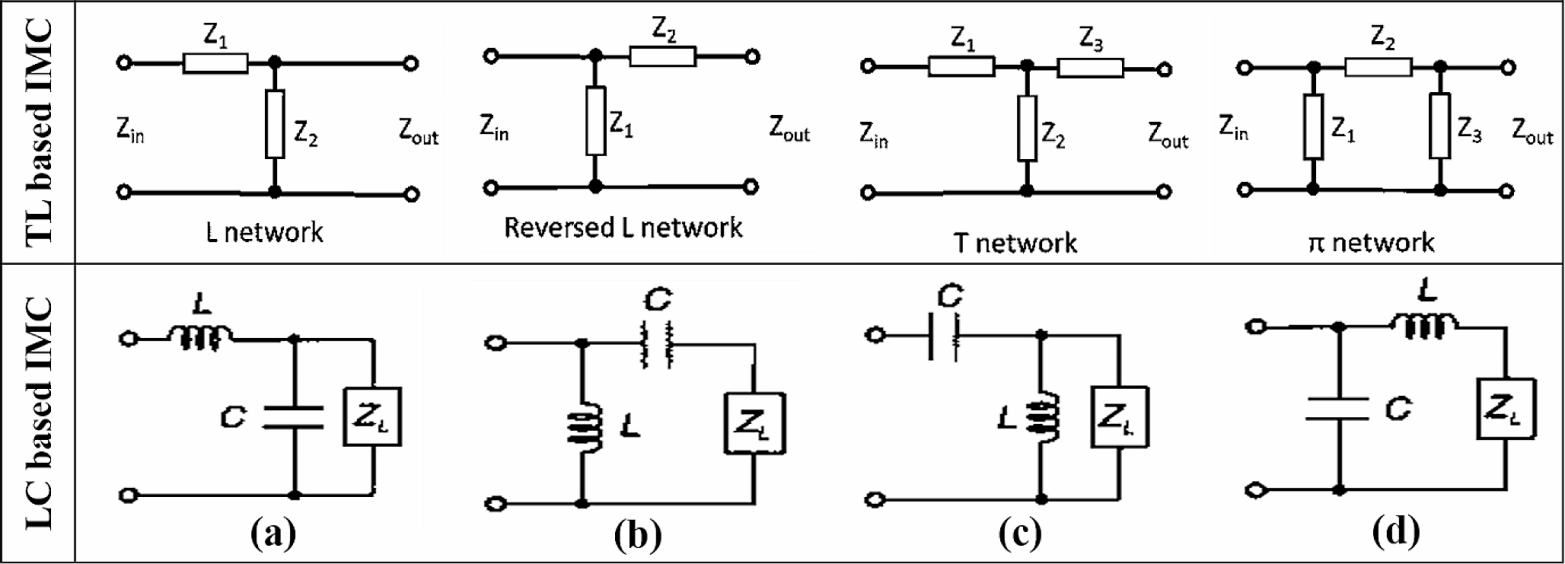
Schematic of various IMC`s configurations: **a** and **d** low pass IMC, **b** and **c** high pass IMC.

The reflection coefficient that can be used to measure the impedance matching depends on reflection coefficient, S_11_, impedance Z_REC_ of the rectifier circuit, and impedance Z_ANT_ of the antenna. Considering the reflection coefficient of -3 dB means that half of the transmitted power is reflected to the RA. S_11_ less than − 10 dB indicates less than 10% power is reflected and the system is considered as optimally matched in antenna communication systems theoretically [59]. In the literature IMC can be accomplished in two ways, one is by using distributed elements based on transmission line (TL) or stubs and the other is by using lumped elements which is LC based circuit, with L, T, and Л matching configurations as shown in Fig. 4. The L matching topology is mostly favored due to its compact size, uncomplicated design, and controllability while the T and Л or a combination of these three topologies can be adopted to boost the harvested voltage by the expense of large size, varying quality factor (Q) and complexity of circuit [60].

Designing an efficient IMC necessitates meticulous attention to optimizing design considerations, evaluating antenna and rectifier impedance, selecting appropriate components, and accounting for environmental variables. Han and Perreault [61] presented an analytical model for the development of an efficient (95%) IMC through optimizing the component values and design consideration. IMC based on lumped elements is simple and adequate to implement, for example, Khan et al. [22] developed single branched L topology and optimized as IMC for RFEH at a higher frequency of 2.45 GHz with − 14 dB reflection coefficient. Similarly, a modified and tunable T network based on lumped elements is designed to achieve impedance matching between the antenna and rectifier [62]. Also, Shah and Yoo [63] achieved the impedance matching only using an inductor between a dual band antenna (915 MHz and 1900 MHz) and rectifier circuit. IMC based on distributed elements uses Microstrip lines, short, open, meandered line, and radial stubs to achieve the impedance matching between RA and rectifier circuits. For example, Liu et al. [64] proposed dual band IMC with a combination of open stubs Л network and shorted stubs L network. The Л configuration is employed to transfer arbitrary impedances that depend on frequency into conjugate impedances, subsequently matched to RA impedance (Z_ANT_) through the utilization of L configuration. The reported IMC used five TL segments to obtain the reflection coefficient of − 38 dB for 915 MHz and − 40 dB for 2450 MHz. Le et al. [65] developed a TL-based distributed elements IMC for two frequency bands (915 MHz and 2450 MHz) that uses only three stepped stubs TL segments in the reversed L configuration to gain the impedance matching between antenna (Z_ANT_) and rectifier with a compact size. Similarly Roy et al. [66] produced a broadband novel IMC to harvest RF energy from a broad RF frequency band (800 MHz to 2600 MHz). The developed IMC is based on hybrid technology that capitalizes lumped elements (four inductors and two capacitors) with the combination of a radial stub to achieve impedance matching of RA and rectifier over the broadband. Furthermore, a complex IMC has been developed with six TL segments [67] and nine TL segments [68], additionally, a tunable IMC [69, 70] has been reported to achieve impedance matching of wideband and broadband RFEHs. Table 5 provides an overview of diverse IMCs, featuring specifications such as network topology, frequency, fabrication method, and reflection coefficient. The design of IMC for RFEH is a tradeoff among various attributes, such as device size, frequency band, adjustability, and circuit complexity. In RFEH for low power devices implementing TL-based matching can suffer the issues of circuit complexity and size. Moreover, Rehman et al. [71] determined the insertion losses of TL and LC-based IMC and it is reported that below 2600 MHz both the IMC network behaves similarly, however, above 2600 MHz, TL-based IMC gains notable advantages over its counterpart LC-based IMC. RF sources with high power density are operating below 2600 MHz (GSM-900, GSM-1800, UMTS-3G, LTE-4G, and Wi-Fi) and the use of TL-based IMC with larger size for such RFEHs is less significant.

**Table 5:** Specification of various impedance matching circuit reported for RFEH

Ref	IMC type	Frequency (MHz)	Fabrication Method	Reflection coefficient (dB)
[22]	LC based L type network	2400	PCB	-14
[72]	TL based L type network	2450	PCB	-38
[73]	TL based T type network	915, 2450	PCB	-24,4, -27.5
[66]	TL based Л and L type network	915, 2450	PCB	-39, -40
[48]	TL based two T type network	1800, 2100	PCB	-14, -16
[74]	LC based two L type network	915, 2450	CMOS	-37.01, -32.7
[75]	Off chip matching network	902, 2450	CMOS	-25, -23
[76]	0ff chip matching network	2400	CMOS	-22.8
[70]	LC based tunable L network	700, 800, 900	CMOS	-36, -23, -10
[77]	LC based L and Л network	750, 1800, 2400, 5800	CMOS	-25, -25, -25, -21

## AC–DC rectifier circuit of an RFEH

**Fig. 5:**
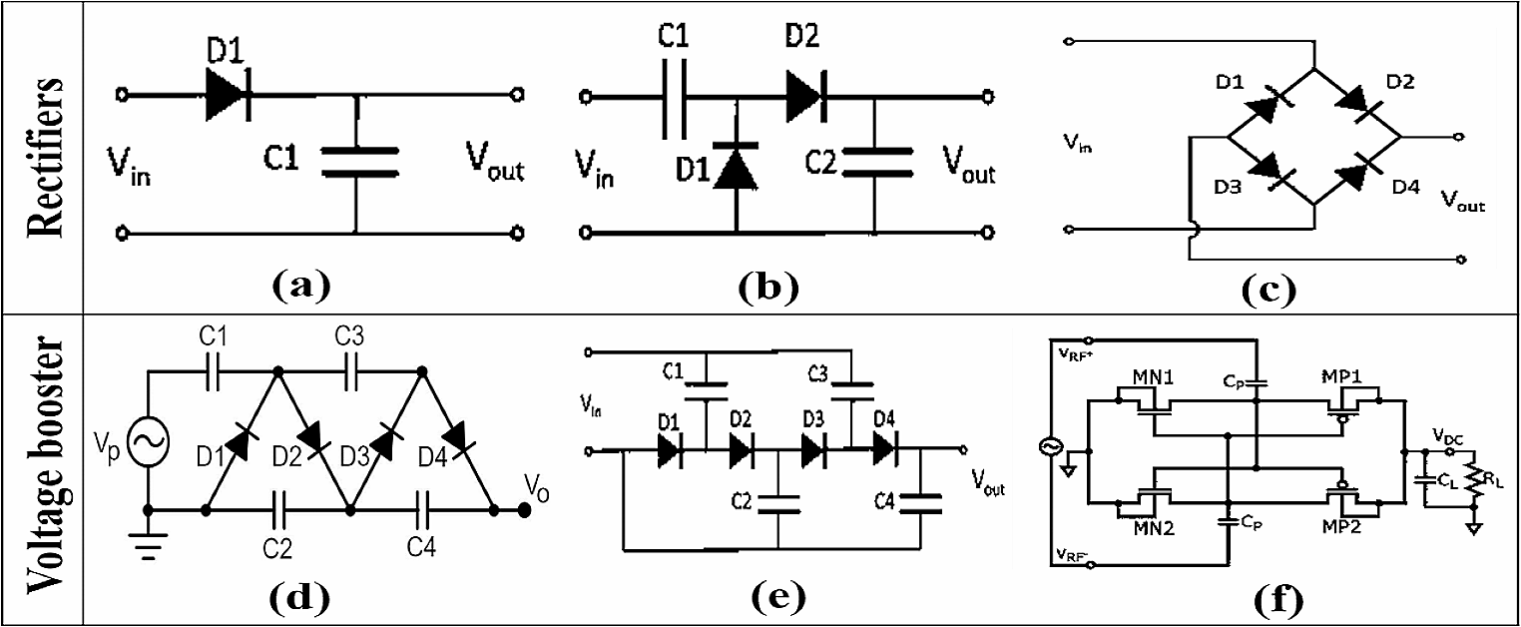
Schematic of different rectifiers and voltage boosters: **a** half wave rectifier; **b** full wave rectifier; **c** bridge rectifier; **d** Cockcroft-Walton rectifier; **e** Dickson rectifier and **f** differential drive rectifier

The RF signals scavenged by RA and transmuted by IMC are in the form of sinusoidal waveform (AC) and narrowband voltage signals with low power density that need to be rectified into DC voltage or could be boosted in order to be utilized for the operation of numerous sensor applications [78]. Rectifiers can be classified as half-wave and full-wave, half-wave rectifiers operate on only one diode which is connected in series or in parallel to load and rectifies only half cycle of the input AC into less efficient DC voltage [79], while full wave rectifiers operate on two diodes with the capability of rectifying full cycle of AC into DC which are more efficient than half wave rectifiers along with circuit complexity. To boost the output voltage in accordance with applications, full wave rectifiers can be designed into various configurations with many numbers of stages, such as, Greinacher or Cockcroft-Walton rectifier, Dickson rectifier and Differential drive rectifier as shown in Fig. 5.

The performance of the rectifier circuit is highly dependent on its power conversion efficiency (PCE) and sensitivity for low-power applications.

6 $${\eta }_{rec}= \frac{{V}_{o}^{2}}{{P}_{in}\times {R}_{L}}\times 100 \left(\%\right)$$

The PEC of rectifiers is a function of the input RF power *Pin*, output DC voltage *Vo* and output load resistance *R*_*L*_ as shown in Eq. 6.

Khan et al. [22] utilized two stages Cockcroft-Walton rectifier with four Schottky diodes (HSMS-2850) to rectify RF signals from single band RA, it is reported that 64% (simulated) PCE is achieved at 0 dBm with 5.2 V output voltage. Greinacher two stages rectifier with Schottky diodes (HSMS-285 C) is developed by Papadopoulou et al. [80] with a dual-band RA and it is reported that high PCE of 85% (simulated) and output voltage of 4.32 V is achieved with a load resistance of 13 kΩ for an input power of 0 dBm. Cockcroft-Walton rectifier uses capacitors in series for two or multiple stages as shown in Fig. 5 which leads to a notable drop with respect to the expected multiplication factor in output voltage after every stage [81]. Dickson rectifier is the modified version of the Cockcroft-Walton rectifier that uses capacitors in parallel to minimize the voltage drop in multi-stage rectifier design, it is also reported that the Dickson rectifier is an appropriate choice for low voltage utilization while the Cockcroft-Walton rectifier is usually implemented for high voltage utilization [82].

7 $${V}_{out1}=2{V}_{p}- {V}_{t1}- {V}_{t2}$$

The output voltage of the single stage Dickson rectifier can be expressed as a function of peak input voltage V_P_, and the threshold voltage levels Vt_1_ and Vt_2_ of the first and second diode/transistor respectively. However, for the Dickson rectifier having multiple stages, output voltage can be expressed as a function of the product of Vout_1_ (output voltage of single stage`s rectifier) and n (number of stages in the rectifiers) as shown in Eq. 8.

8 $${V}_{out,n}=n {V}_{out1}$$

Shah and Yoo [63] implemented a two-stage Dickson rectifier with Schottky HSMS-2860 is implemented for dual band RA and it is reported that PCE of 82% is obtained for the input power of 2 dBm having load resistance of 15kΩ. Similarly, Basir et al. [83] implemented two-stage Dickson rectifier to achieve a higher PCE of 89% with an output voltage of 3 V for the input power of 15 dBm. The reported rectifier topologies [22, 63, 83] utilized Schottky diodes for rectification purposes, as such diodes are developed for low voltage applications at a frequency range between 915 MHz to 5.8 GHz and achieved better performance with the qualities of high forward voltage, low resistance and very low capacitance [63]. In accordance with Eq. 8, to increase the level of output DC voltage it is essential to increase the number of stages in the rectifier which leads to huge voltage drops and degrades the overall PCE and sensitivity of the rectifier [82].

The differential drive is a full wave rectifier topology that uses two types of MOSFETs, NMOS and PMOS transistors instead of diodes to minimize the voltage drop and enhance the overall PCE [82, 84]. Stoopman et al. [39] developed a five-stage differential rectifier based on CMOS and it is reported that for an input of -17 dBm 40% PCE is obtained with an output voltage of 1 V. To reduce the leakage current and threshold voltage of MOSFETs in differential rectifiers, a bootstrapped capacitors mechanism based on CMOS is proposed [86] for low power applications. It is reported that by adopting a bootstrapped mechanism PCE of 80% is achieved with an output voltage of 2 V for 2 kΩ load resistance. Table 6 offers an overview of reported rectifier variants, including details on the number of stages, the specific diodes or transistors utilized, and the highest attainable PCE. A single-stage full-wave differential rectifier employs four transistors, resulting in a larger overall size, yet it delivers with better performance in high-frequency RFEH system [95]. Conversely, a single-stage full-wave Dickson rectifier can be realized with just two diodes, though it comes at the expense of reduced efficiency and a lower output voltage when compared to the differential rectifier. The decrease in efficiency may necessitate the utilization of multiple stages in the Dickson rectifier to achieve the desired performance. Nevertheless, compact two-stage Dickson rectifiers designed for WPT application in IMDs have demonstrated remarkable performance. These rectifiers achieved PCE of 82% at 2dBm [63] and 89% at 15dBm [83].

**Table 6:** Characterization of reported rectifier circuits for RFEH

Ref	Rectifier type	Number of stages	Number of branches	Diodes/Transistors	PCE (%)
[87]	Half wave	Four	One	MOSFET	37.4
[65]	Half wave	One	Two	BAT 15-03 W	74
[88]	Half wave	One	Two	SMS 7621	64.5
[89]	Half wave	One	Two	HSMS 2860	82.5
[90]	Full wave	One	Two	SMS 7630	65
[91]	Full wave	One	Two	HSMS 2822	71
[73]	Full wave	One	Two	HSMS 2862	81.7
				SMS 7630	69.2
[92]	Cockcroft Walton	Seven	One	HSMS 285 C	17
[93]	Cockcroft Walton	Eight	One	HSMS 2850	25
[63]	Dickson	Two	Two	HSMS 2860	82
[83]	Dickson	Two	One	SMS 7630	89
[94]	Differential drive	Four	Three	MOSFET	65
[95]	Differential drive	Three	One	MOSFET	73
[39]	Differential drive	Five	One	MOSFET	40

## Comparison and discussion

This section conducts a comparative analysis and discussion of the performances of receiving antennas, matching circuits, and rectifiers developed for RFEHs. This assessment takes into account metrics such as output voltage, output power, and system PCE as shown in Table 7. It is reported that the majority of RFEHs are developed for GSM-900, GSM-1800, UMTS-3G, and LTE-4G as an enormous number of mobile phone base stations deployed all over the world operate with such frequency bands. The development of a foremost RFEH is immensely dependent on the selection of the RA, multiband and high gain RAs are capable of receiving maximum RF energy residue from the transmitters that will lead to higher output power.

**Table 7:** Performance and specifications of reported RFEHs

Ref	RA dimension (mm)	Receiving antenna type	Resonating frequency (MHz)	IMC type	Rectifier circuit type	Output Voltage (V)	Output Power (dBm)	PCE (%)
[22]	57.3 × 50.5 × 1.6	Microstrip patch	2400	LC-based	Dickson	1.5	-123 @ 150 m*	64
[38]	96.3 × 96.3 × 1.7	loop	915, 945	TL based	DD^5^	0.23	-16 @ 915 MHz	65
[48]	190 × 100 × 62	Quasi Yagi	1800, 2100	TL based	HW^2^	0.48	-20 @ -16^1^	30
[49]	240 × 240 × 2	Microstrip patch	877–998	LC-based	FW^3^	2.78	5 @ 17.1^1^	6.1
[96]	304 × 304 × 1.6	Quasi isotropic patch	470–608	LC-based	FW^3^	3	-6.3 @ 5800 m*	28
[97]	110 × 85 × 1.6	E-shaped patch	840–1170	TL based	CCW^4^	2.9	-17.21 @ 50 m*	-
[98]	28.5 × 28.1.57	Co-planar waveguide	2450	TL based	CCW^4^	2	-57.3 @ -50^1^	20
[99]	77.6 × 40 × 0.1	Dipole	464	LC-based	HW^2^	2.3	21.6 @ 33.4^1^	6.5
[100]	40 × 59 × 1.6	Folded dipole	900, 2450	TL based	Dickson	2.75	-21.2 @-16^1^	30
[101]	180 × 146 × 1.6	Microstrip patch	900	LC-based	Dickson	2.1	-20 @ 10^1^	0.1
[102]	200 × 200 × 1.52	Patch	1800, 2100, 2450	TL based	HW^2^	0.39	-12 @ -7.1^1^	40
[103]	40 × 40 × 1.6	Fractal patch	2100, 5800	TL based	HW^2^	4.52	4.3 @ 5^1^	86
[104]	82 × 94 × 1.6	Triangular monopole	950	-	FW^3^	1.26	7.21 @ 1.5 m*	60
[105]	70 × 70 × 1.6	Cross dipole	1800–2500	TL based	CCW^4^	0.29	-12.5 @ -10^1^	55
[106]	40 × 40 × 1.6	Triangular monopole	2250–2750	TL based	DD^5^	6.58	4 @ 5^1^	80
[107]	60 × 60 × 1.6	Co-planar waveguide	5800	-	FW^3^	0.45	-7.3 @ -6^1^	73.4

*meters, ^1^simulated-result, ^2^Half-wave, ^3^ Full-wave, ^4^Cockcraft-Walton, ^5^ Differential-drive

**Fig. 6:**
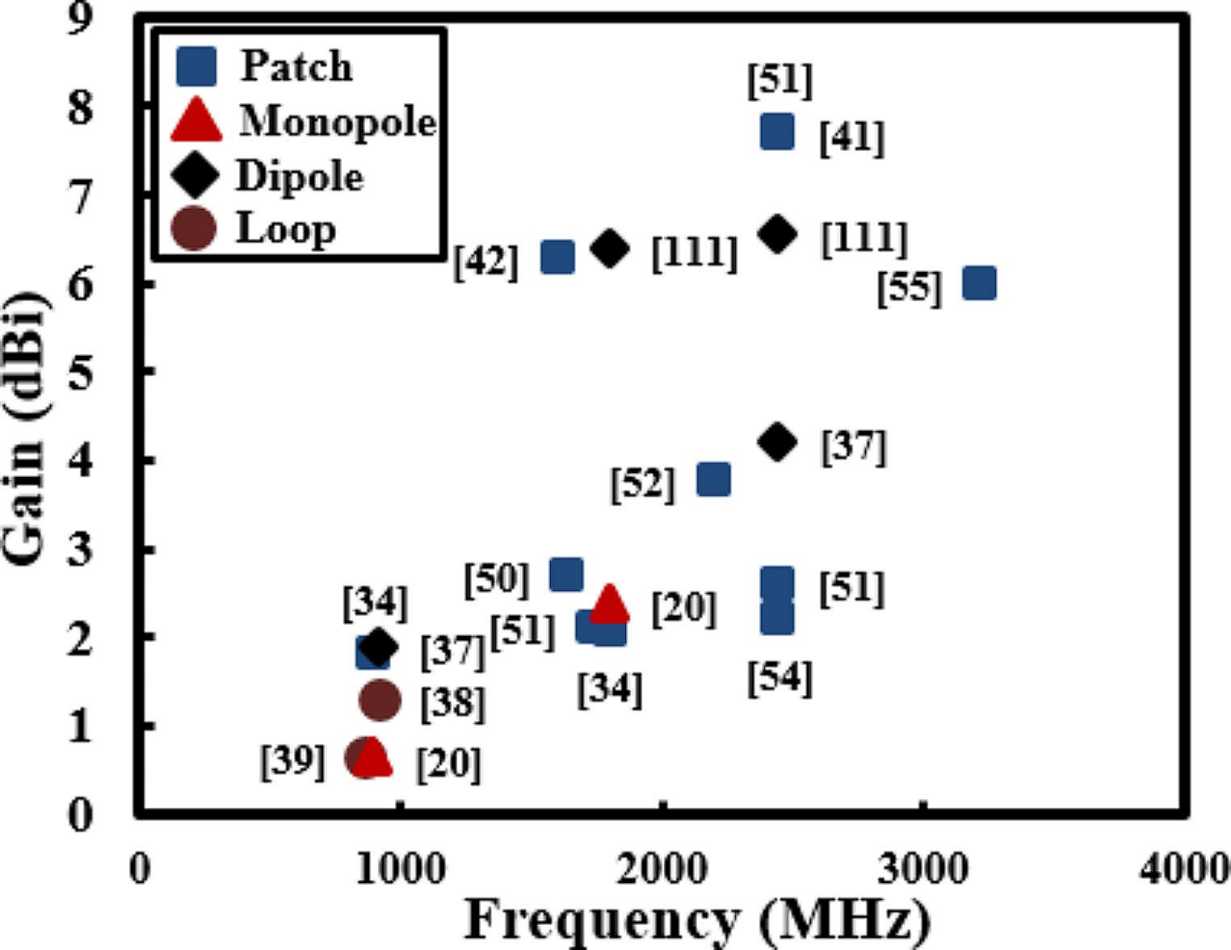
Gain of RAs as a function of resonating frequencies

Figure 6 compares various miniaturized RAs developed for RFEHs. Stoopman et al. [39], proposed compact rectangular loop RA measuring dimensions of 1200 mm^2^ but it exhibits a modest gain of 0.65 dBi. Likewise, dual-band RA characterized by its substantial gain of 6.53 dBi is reported [110] but notably, this particular RA is associated with larger physical footprints, measuring 11,400 mm^2^. Improving the performances of RA in terms of gain and efficiency while maintaining the compact size can pose challenges due to the intrinsic physical limitations of antennas. Nevertheless, various miniaturized strategies coupled with careful design and optimization can be embraced to achieve a harmonious balance between compact size and enhanced performances tailored to a specific application and frequency band. To utilize RFEHs for wireless sensors and IMDs, miniaturized sizes of RAs can be obtained through various techniques and geometries, such as slotted [50, 51], fractal [52, 53], meandered lines [54, 55], spiral [108] and conformal [109]. Similarly high RAs performance, efficiency, gain, and radiation pattern can be achieved through implementation of several array elements [111], also circular polarization [112] holds worthy importance in the domain of RFEH as such devices encounter signals transmitted from unidentified and diverse sources in the environment.

**Fig. 7:**
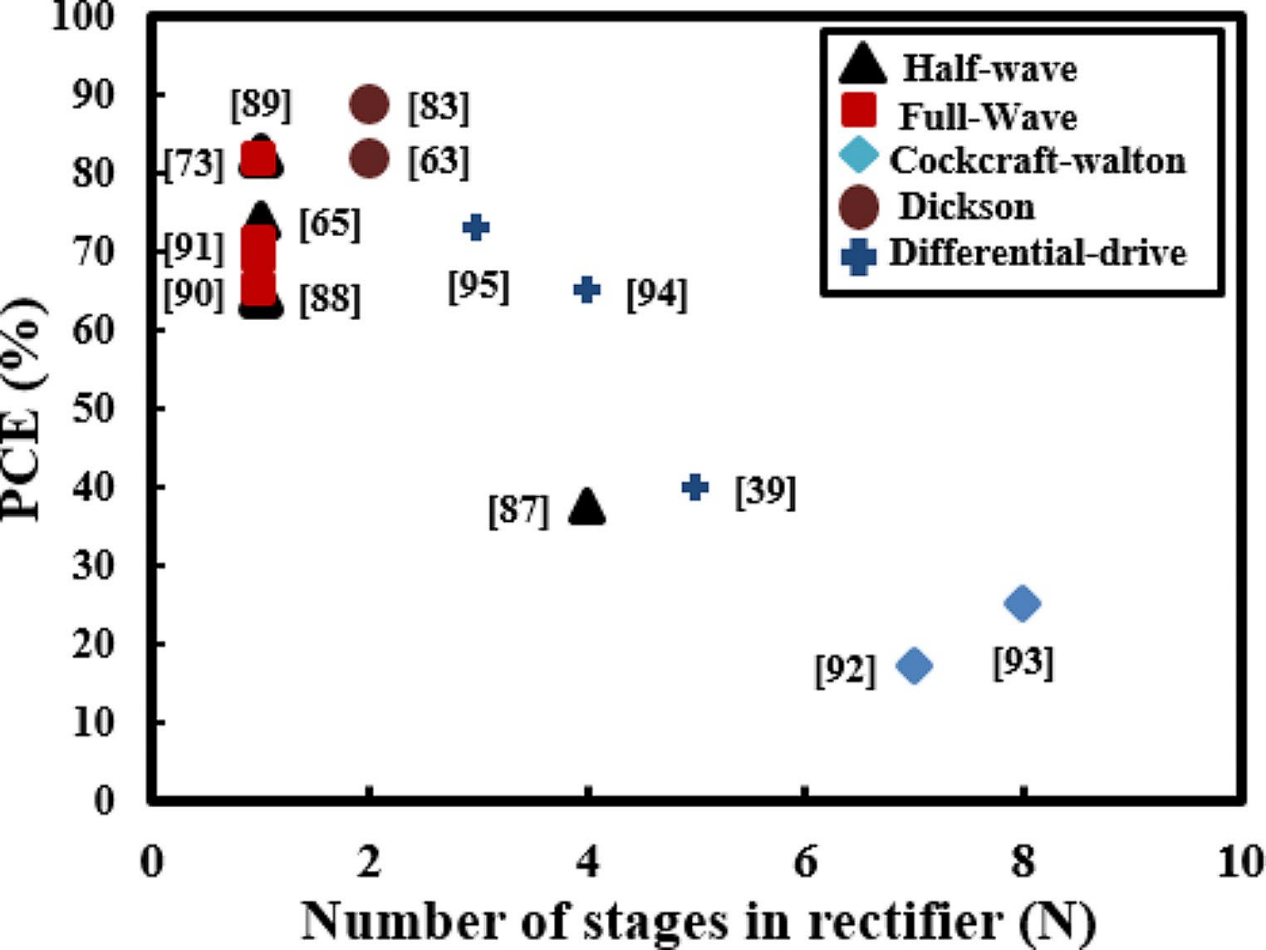
PCE (%) against number of rectifier’s stages (N)

The performance of AC-DC rectifiers in terms of PCE and sensitivity has been the spotlight of the researchers recently as the overall efficiency of RFEHs is particularly dependent on the rectifier’s performance. Figure 7 compares the PCE of various rectifiers reported in the literature. It can be seen that CMOS based differential drive rectifiers are capable of obtaining maximum efficiency by controlling voltage drops and leakage current [82, 84, 86] with comparably large sizes, also rectifiers with higher stages can achieve maximum output voltage levels at the cost of large sizes and low PCE [85, 93, 94]. IMDs and wireless sensor based devices are primarily low-power devices as discussed in Table 1, using full-wave rectifiers with the fewest possible stages is highly advantageous when aiming to achieve maximum PCE and minimize the overall size of the system [63, 83].

**Fig. 8:**
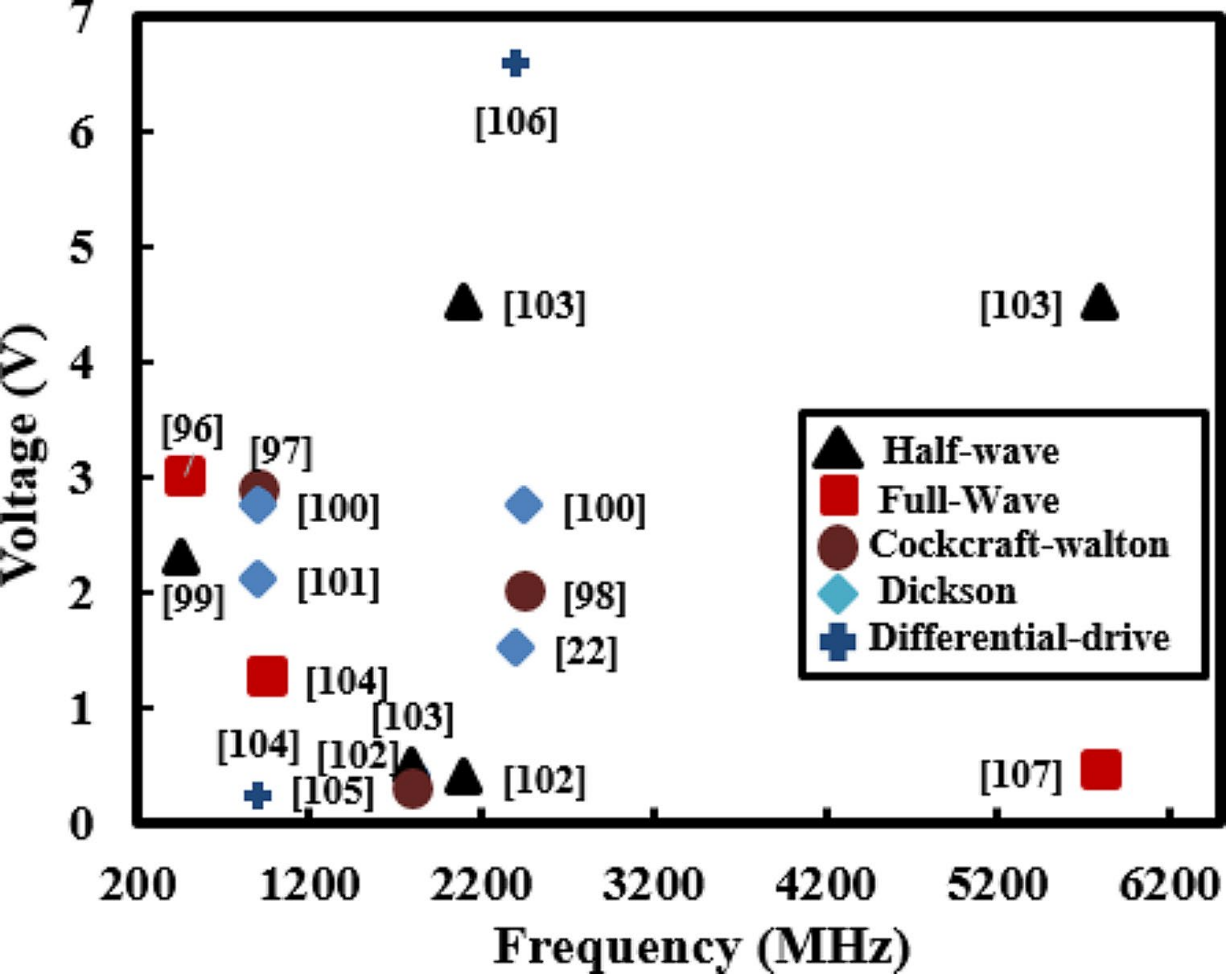
Output voltage as a function of frequency

The output voltage of different rectifiers as a function of frequencies is shown in Fig. 8. Zengin et al. [36] developed an RFEH system that employs a loop RA and differential drive rectifier, achieving maximum output voltage of 2.31 V from GSM-900 transmitter. Similarly, RFEH developed with a wideband quasi-isotropic patch RA is capable of harvesting a maximum voltage of 1.2 V at a long distance of 5800 m from the TV and FM transmitters [96]. RFEH featuring an E-shaped patch RA and a full-wave rectifier, obtained a peak harvested voltage of 2.9 V when receiving energy from a GSM-900 transmitter located 50 m away [103]. The voltage levels obtained as indicated [97–100], surpass the 2 V threshold. This holds significant potential for their use in low-voltage applications such as wireless sensors and IMDs as outlined in Table 1.

**Fig. 9:**
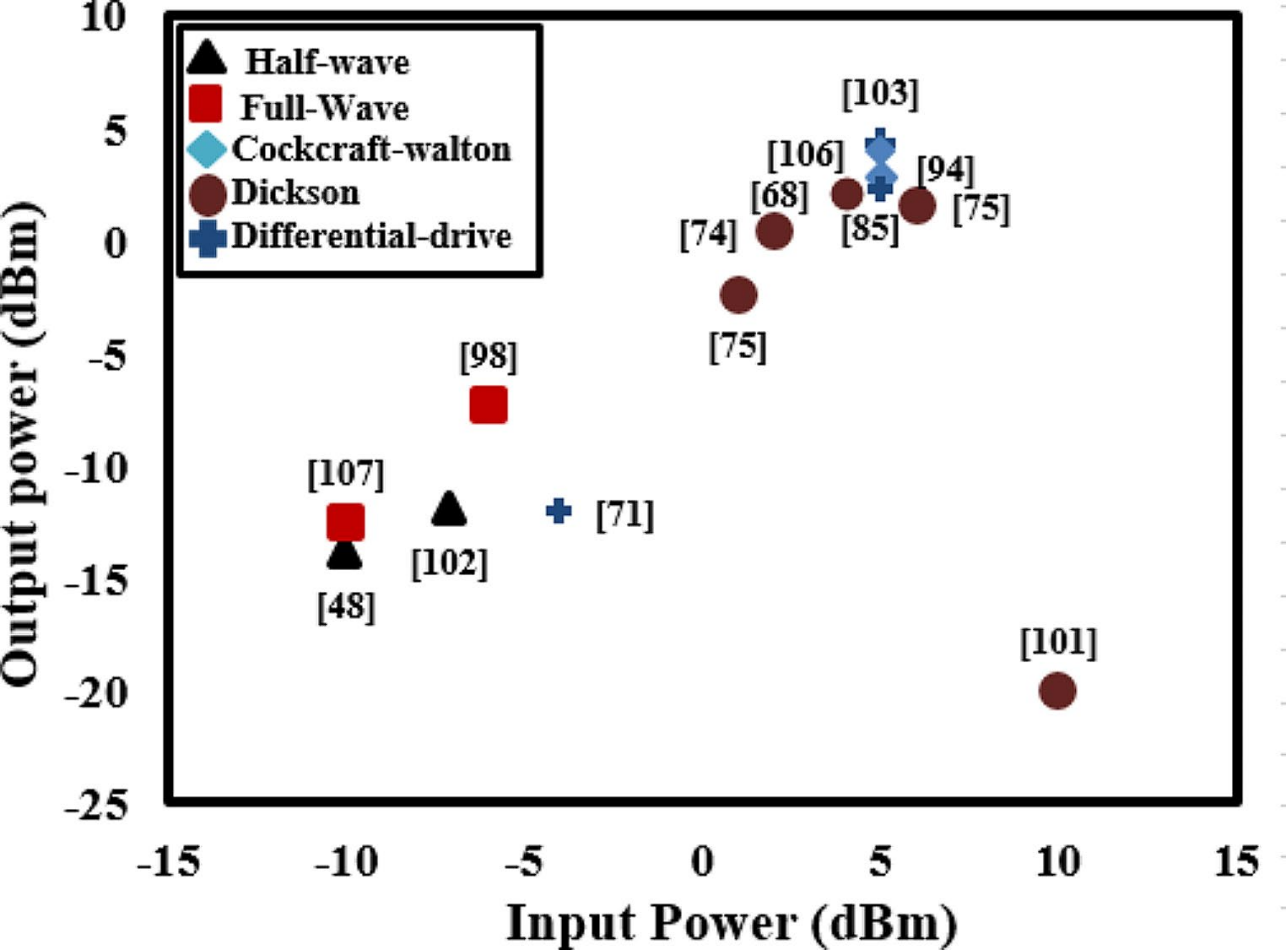
Output power as a function of input power

The output power of RFEHs as a function of frequency is shown in Fig. 9. RFEH system employing a wideband isotropic RA, demonstrates the capability to harvest − 6.3 dBm power from the transmitter located at a distance of 5800 m [96]. The reported system can be considered a viable choice for powering IMDs, given the compact geometry of the harvester. Likewise, another RFEH featuring an E-shaped RA harvested output power of -17.2 dBm from GSM-900 transmitter located at 50 m away [97]. This system can be regarded as a feasible choice to supply power for electronic watch and glucose sensors, aligning with their power requirements. Arrawatia et al. [104] developed RFEH with triangular monopole RA, obtained high efficiency and employed the capability of harvesting 7.2 dBm power from the GSM-900 transmitter located at 1.5 m away. The reported RFEH offering a practical and feasible opportunity for powering temperature sensors, smoke detectors, and low power IMDs, aligning with their specific power requirements as outlined in Table 1.

## Future prospects and challenges of RFEHs

The field of RFEH poised to encounter many future challenges and opportunities across diverse dimensions, including power and voltage generation, size reduction, fabrication methodologies, and adaptability for implantable and wearable devices.

The primary challenge facing RFEH lies in augmenting power generation efficiency. Researchers are actively engaged in enhancing antenna designs, refining impedance matching techniques, and optimizing rectifier efficiency to elevate the overall efficiency of the system. A notable challenge in the field of RFEH is the harvesting and regulation of specific voltage levels, a critical requirement for low-power devices, notably those employed in IoT applications. To keep pace with the prevailing trend of smaller and compact micro and nano technologies, it is important to reduce the size of RFEH. To accomplish this, utilization of miniaturization techniques, implementation of advanced materials, and exploration of inventive antenna designs are essential steps to minimize the physical footprint of RFEHs. The implementation of efficient, cost-effective, and versatile fabrication methods is vital for advancing RFEH technologies, making them more accessible and commercially viable. Emerging approaches, such as, 3D printing and flexible electronics hold significant promise in this domain. The integration of RFEH with efficient energy storage solutions, such as, super-capacitors and advanced batteries holds great significance. This integration guarantees a consistent power supply to applications, even in the case of RF energy fluctuations or when the ambient RF sources energy levels are weak. Exploring the implementation of RFEH in biocompatible and implantable devices holds significant potential. Nevertheless, there are concerns surrounding the assurance of safety, reliability, and longevity for wearable devices and medical implants. The utilization of RFEH has the potential to bring about an interesting revolution in the field of wearable devices. Nonetheless, challenges remain in the fabrication of flexible and adaptable antenna designs, as well as in effectively addressing concerns related to comfort and aesthetics in the design of wearables. Prioritizing and emphasizing adaptability and resilience is essential for RFEH to guarantee consistent operation in a challenging and diverse environment marked by issues like interference, reflections, and extreme temperature fluctuations. Creating industry-wide standards for RFEH technologies is necessary. These standards play a pivotal role in promoting interoperability, ensuring that devices from diverse manufacturers can effectively manage and utilize RF energy.

Overcoming the challenges outlined above requires seamless interdisciplinary cooperation among designers, engineers, and dedicated researchers striving to advance RFEH systems. With the continuous advancement of technology, RFEH stands poised to fulfill a crucial role in providing power to low-power IoT devices, wearable electronics, and sensors.

## Conclusion

This work delves into the latest state of the art of RFEHs developed for low-power sensors and implantable devices. In the field of RFEH, an in-depth review of recent developments in receiving antenna design is presented, to improve performances (gain and efficiency) while simultaneously achieving compact and miniaturized sizes. Furthermore, a thorough review of techniques for improving performance and achieving miniaturization is conducted, encompasses a range of antenna geometries, including those employing slotted, meandered lines, fractal patterns, spirals, and conformal designs, which are extensively reported in the literature. Additionally, performances and geometries of various impedance-matching circuits are compared and reviewed, including both lumped and distributed element-based designs, reported for antenna and rectifier applications. This analysis aims to identify implicit and appropriate matching circuits suitable for RFEHs. In the pursuit of RFEH for low-power sensors and implants, different RF-DC rectifiers and voltage boosters are explored and compared to assist in the selection of the most appropriate option for specific applications. Finally, this study identifies potential future challenges and opportunities in the domain of RFEH, encompassing diverse aspects such as power and voltage generation, size reduction, fabrication methods, and adaptability for implantable and wearable devices.
